# Using the media social Facebook to increase the community voluntarism and engagement to monitoring ARV in Indonesia

**DOI:** 10.1186/2052-3211-7-S1-P10

**Published:** 2014-12-17

**Authors:** Irwandy Widjaja, Aditya Wardhana, Budi Rissetiyabudi Darma Adi, Sindi Putri, Ayu Oktariani, Setio Budi Deni Widodo

**Affiliations:** 1 Indonesia AIDS Coalition (IAC), Jakarta, Indonesia

## Background

In Indonesia issues of late deliveries and expiry of antiretroviral (ARV) medicines are significant. Indonesia with 7 main islands and 14.000 islands has a number of distribution difficulties. In addition there is a lack of appropriately qualified personnel handling ARV logistics. Seeing this situation the Indonesia AIDS Coalition (IAC) sought to initiate ARV monitoring through social media (Facebook) in 2011. Within the Indonesian there are 75 million internet users and 62 million who use Facebook. The Facebook community used to oversee the availability of ARV is called “Monitoring ARV” with 384 members including: people living with HIV in the community, doctors, professionals, activists and non-government organisations working in AIDS response.

## Method

In the beginning the Monitoring ARV Facebook Group only consisted of ten people, quickly growing to 384 members. In the absence of funding the socialization around this group has only spread by social media and other organizational activities or in meeting activities with other stakeholders. ARV stock out reports are received by Facebook group members and are then reported to the Ministry of Health, Sub directorate AIDS through e-mailing a Facebook screenshot, but only after they are verified. These reports are then followed up by the IAC.

## Results

Since this project began there has been improved two way communication between the IAC and the AIDS sub directorate. Before this project medicines delay problems took 15-25 working days to solve but since the instigation of Monitoring ARV these problems are resolved in approximately seven working days. The Monitoring ARV project has increased the community voluntarism and engagement to complete ARV monitoring. Through Monitoring ARV communities are reminded to always check the medicine quality, amount received, packaging and expired date as this knowledge is limited within the community.

## Discussion

We can see that there is two way communication between community and government in securing ARV medicines availability. This has not happened before. There is discretion from the community to do the reporting through the Monitoring ARV in Facebook without unhindered bureaucracy. This approach is has been quiet economical, especially when considering the geographical challenges of Indonesia. Community engagement through voluntarism has resulted in a shared responsibility for monitoring ARVs.

## Lessons learned

The problem of ARV availability can be solved with good cooperation and communication between community and government. The community engagement in supply chain management of ARVs is very important. Community engagement could be extended from the national level to the district level through voluntarism.

